# A specific gene expression signature for visceral organ metastasis in breast cancer

**DOI:** 10.1186/s12885-019-5554-z

**Published:** 2019-04-08

**Authors:** C. D. Savci-Heijink, H. Halfwerk, J. Koster, H. M. Horlings, M. J. van de Vijver

**Affiliations:** 1 Amsterdam UMC, University of Amsterdam, Department of Pathology, Meibergdreef 9, 1105 AZ Amsterdam, the Netherlands; 2 Amsterdam UMC, University of Amsterdam, Department of Oncogenomics, Meibergdreef 9, 1105 AZ Amsterdam, the Netherlands; 3 The Netherlands Cancer Institute, Department of Pathology, 1066 CX Amsterdam, the Netherlands

**Keywords:** Organotropism, Pathology, Site-specific relapse, Microarray

## Abstract

**Background:**

Visceral organ metastasis is associated with poor survival outcomes in terms of metastasis free- and overall survival in breast carcinomas. Identification of a gene expression profile in tumours that selects a subpopulation of patients that is more likely to develop visceral organ metastases will help elucidate mechanisms for the development of distant metastases and could be of clinical value. With this study we aimed to determine genomic predictors that would help to distinguish breast cancer patients with more likelihood to develop visceral metastasis.

**Methods:**

Gene expression profiling data of 157 primary tumours from breast cancer patients who developed distant metastases were analyzed and differentially expressed genes between the group of tumours with visceral metastasis and the those without visceral metastases were identified. Published data were used to validate our findings. Multivariate logistic regression tests were applied to further investigate the association between the gene-expression-signature and clinical variables. Survival analyses were performed by the Kaplan-Meier method.

**Results:**

Fourteen differentially expressed genes (*WDR6*, *CDYL*, *ATP6V0A4*, *CHAD*, *IDUA*, *MYL5*, *PREP*, *RTN4IP1*, *BTG2*, *TPRG1*, *ABHD14A*, *KIF18A*, *S100PBP* and *BEND3*) were identified between the group of tumours with and without visceral metastatic disease. Five of these genes (*CDYL*, *ATP6V0A4*, *PREP*, *RTN4IP1* and *KIF18A*) were up-regulated and the other genes were down-regulated. This gene expression signature was validated in the training and in the independent data set (*p* 2.13e− 08 and *p* 9.68e− 06, respectively). Multivariate analyses revealed that the 14-gene-expression-signature was associated with visceral metastatic disease (*p* 0.001, 95% CI 1.43–4.27), independent of other clinicopathologic features. This signature has been also found to be associated with survival status of the patients (*p* < .001).

**Conclusion:**

We have identified an unique gene expression signature which is specific to visceral metastasis. This 14-gene-expression-signature may play a role in identifying the subgroup of patients with potential to develop visceral metastasis.

**Electronic supplementary material:**

The online version of this article (10.1186/s12885-019-5554-z) contains supplementary material, which is available to authorized users.

## Background

The implementation of breast cancer screening programmes and improved options for the treatment of patients with early breast cancer have contributed to the improved outcome in breast cancer [1]. However, once metastatic disease develops, breast cancer is still a deadly disease [2, 3]. Predicting the likelihood of metastatic behaviour and organ-specific metastasis of the primary tumours could help to improve the modalities for the treatment of the primary tumour and of metastatic disease.

The relationship between primary tumour and their metastases has been an important area in cancer research since the “seed and soil’ theory proposed by *Stephen Paget* [4]. Several studies have investigated the predictors for metastatic potential [5–7] of the primary tumours and site- specific distant organ metastasis [8–11] in breast cancer. Some of these studies using animal models and genomic profiling have identified gene expression signatures that were associated with organ specific metastasis. In particular, using an experimental system based on the in vivo selection of MDA-MB231- derived breast cancer cell lines with specific organotropism, Massague and co-workers have identified genes related to bone, lung and brain metastasis. They have suggested that whereas some genes may determine a breast cancer’s overall classification and prognostic signature, some other superimposed tissue-specific metastatic gene expression profile(s) in a subset of tumour cells may affect the cancer’s organ specific metastatic behaviour [12].

It has been demonstrated that metastases and primary breast cancers show similar gene expression profiles [13–15]. We previously reported gene expression profiling experiments performed on primary breast cancers to identify gene expression profiles for organ-specific metastasis. We have recently presented a novel 15-gene expression signature for bone specific metastasis in breast cancer [16]. This new gene expression signature was found to be associated with the likelihood of bone metastasis development in ER-positive and ER-negative tumours, in the training set as well as in an independent data set including 376 tumours with known clinical metastatic disease. We have shown that 80.5% of the patients with luminal subtype tumours developed bone metastasis as opposed to, respectively, 41.7 and 55.6% of the basal type and HER2-like tumours (*p* 0.001). We have also identified that 70.4% of luminal type tumours, 87.5% of basal type tumours and 77.8% of HER2-like tumours developed visceral organ metastasis (liver, lung and brain). Among basal type tumours 66.7% developed visceral metastasis as first metastasis site and 29.2% of these tumours had only visceral metastasis during the course of disease. Survival analyses revealed that patients who developed visceral metastasis had worse survival outcome, in terms of metastasis specific survival and overall survival and they frequently developed multiple metastasis during the course of the disease [17]. In this study we sought a gene expression profiling identifier to select the subgroup of tumours that are most likely to develop visceral organ metastasis.

Here we present a gene-expression signature which is found to be associated with development of visceral organ metastasis in breast carcinomas.

## Methods

### Patients and tumour samples

One hundred fifty-seven primary breast carcinomas from patients who all developed distant metastases were included in this study. This series of tumours has been described previously [17]. The national ethical guidelines of ‘Code for Proper Secondary Use of Human Tissue’ developed by Federation of Medical Societies (FMWV) in the Netherlands were followed for this study [18].

Clinical data with detailed information on metastatic behaviour, metastasis site and survival outcomes were collected from the clinical charts for 151 patients as previously published [17]. Briefly, metastasis site was carefully recorded and classified into ever versus never, first versus not first and only versus not only for each organ site. In addition, data on systemic treatment (chemotherapy, hormonal therapy and targeted therapy) used to treat primary and metastatic disease was also available for a subset of patients (*n* = 142 and *n* = 122, respectively). Tumours were evaluated and histological and immunohistochemical characteristics were assessed as previously published [17].

### Gene expression profiling and human breast tumour microarray data sets

The methods for extraction, amplification and hybridization of RNA have been already explained [16]. Detailed information on these techniques is accessible via Illumina website (website (http://www.illumina.com). As described, the arrays were processed in the Central Microarray Facility of the Netherlands Cancer Institute. The data was normalized using robust spline normalization (rsn) and log2 transformed, followed by ComBat (http://www.bu.edu/jlab/wp-assets/ComBat/Abstract.html) to adjust for batch effects. Next to this already published gene expression profiling data set of 157 primary breast tumours a combined data set (GSE2034, GSE12276, GSE2603 and the NKI295 (*microarray-**pubs.stanford.edu**/wound_NKI/Clinical_Data_Supplement.xls*) captured from public domain was used [19]. A subset of tumours (*n* = 376) with clinically proven metastatic disease was utilized for the analyses [19].

### Microarray data analysis

All data were analyzed using the R2 (Microarray Analysis and Visualization Platform) web application, which is publicly available at http://r2.amc.nl. The tumours were also designated to have a “good prognosis” or a “poor prognosis” profile based on the 70-gene prognostic signature as described previously [16].

To validate already published gene expression signatures for lung metastasis [10, 11] and brain metastasis [8], the indicated genes were mapped to Illumina platform via Gene Symbol ID. As previously described [16], respectively a K-means method was used to cluster the patients in 2 groups and a t-test revealed the performance of these signatures in our dataset.

### Identification and validation of site-specific metastasis signature

To identify a gene expression signature associated with organ specific metastasis, we used the one-way ANOVA function in R2. Only the genes with an expression level above background level were included in the analysis (total 16,051 genes). Samples were split into 2 groups; one group in which the patient developed a metastasis and another group in which a patient never developed a certain organ metastasis. The genes that showed a significant differential expression between these groups (*p* value,0.0001) were included in the signature. The metastatic signature was subsequently validated in multiple datasets using the K-means and t-test function in R2. To further investigate the link between this gene signature and clinical variables multivariate logistic regression tests were applied using SPSS Statistics for Windows (Release version 21.0;IBM Corp.2012, Armond, NY). Overall survival and metastasis free survival were analyzed by the Kaplan-Meier method in the training data set. Due to missing survival data in the publicly available files, additional survival analyses were not conducted in the independent dataset. All statistical tests were two sided and *p <* 0.05 was considered to be statistically significant.

## Results

From 157 primary invasive breast cancer from patients who developed distant metastases during follow-up, mRNA expression signatures were assessed using micro array analysis. The patient and tumour characteristics have been described previously [16].

Tumours were subdivided into molecular subtypes with the help of the PAM50 classifier [20]. The distribution of metastatic behaviour including site of metastasis, metastasis timeline and survival outcomes among the molecular subtypes have been published previously and are summarized in Table 1. Adjuvant systemic therapy was administered for 79.4, 72.5, 78.6 and 87.5% of the patients with Luminal A, Luminal B, HER2-like and basal type tumours; respectively. Targeted therapy, by means of trastuzumab, was given to a subgroup of patients (*n* = 10) in the metastatic setting. The tumours were also subdivided into two groups as having “good prognosis” and “poor prognosis” based on their 70-gene expression profile.

**Table 1:** Distribution of metastatic behavior among molecular subtypes

		bone metastasis		visceral metastasis		metastasis timeline^a^		time to develop metastasis^b^	time to last event^c^
		yes	no	yes	no	early	late		
Molecular subtype	Luminal A	54	11	41	24	48	18	45	37
	Luminal B	33	10	35	8	33	10	41	40
	HER2 like	10	8	14	4	15	3	35	21
	Basal type	10	14	21	3	20	4	26	21
	Normal like	1	0	1	0	1	0	10	1
		*p* .001		*p* .090		*p* .749		*p* .052	*p* .001

^a^cut-off point, 5 years

^b^time from surgery date to the development of first metastasis in months

^c^time from development of first metastasis to the last event (death/last follow-up) in months

### Validation of previously identified gene signature (s) for lung and brain specific metastasis

We have investigated three previously published gene signatures for lung [10, 11] and brain metastasis [8].

The gene expression signature (consisting of 54 genes) predicting lung metastases identified by *Minn* et al. [11] identified 55 primary tumours as having a “lung metastasis” gene expression signature. Out of the 55 tumours which positively tested for this signature, 17 (30.9%) were found to have developed lung metastases. Of negatively tested primary tumours, 61 (63.5%) had no lung metastasis (*p* 0.594). When separated according to ER status, of 29 positively tested ER-positive tumours 6 (35.3%) and of 26 positively tested ER-negative tumours 11 (42.3%) developed metastatic disease to the lung (*p* 0.165 and *p* 0.368, respectively). These results show that the 54 gene lung metastasis signature did not predict the development of lung metastases in our patient series. 70.8% of the basal type tumours, 44.4% of the HER2-like tumours and 27.7% of the luminal type tumours was positive for the lung metastasis associated gene expression signature.

The six-gene expression lung metastasis associated signature of *Landemaine* et al. [10] was present in 23 tumours. Nine (39.1%) of these patients with positively tested tumours had lung metastasis, whereas of 128 patients with negatively tested tumours 85 (66.5%) had no metastatic disease to lung (*p* 0.638).

87.5% of the basal type tumours and 0.9% (*n* = 1) of the luminal type tumours was positive for the signature. None of the HER2-like tumours were positive. The 17-gene expression signature of *Bos* et al. for brain specific metastasis was tested as positive in 56 tumours. Sixteen (28.6%) of the patients with positively tested tumours developed brain metastases and 79 (83.2%) of the patients with negatively tested tumours did not develop brain metastases (p 0.102). When the tumours which were positively tested for 17-gene signature [8] grouped according to ER-status, 11 (44%, *n* = 25) of ER-positive tumours and 12 (38.7%, *n* = 31) of ER-negative tumours had brain metastasis (*p* 0.795 and *p* 0.74, respectively). All basal type tumours, 33.3% of the HER2-like tumours and 24.1% of the luminal type tumours were positive for the brain metastasis associated gene expression signature.

### Supervised classification of visceral organ metastasis related genes

**Fig. 1:**
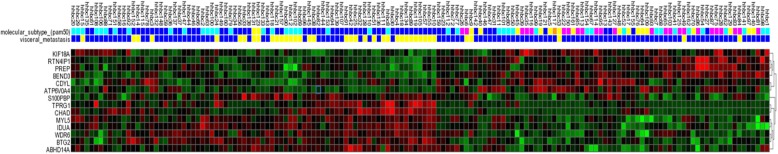
The gene expression pattern of 14-gene expression signature for visceral metastasis in breast cancer. Heat map displays the gene expression profiling pattern of the 14 differentially expressed genes among 151 tumours. Primary tumours of the patients who developed visceral metastasis are illustrated in blue and the ones without visceral metastatic disease are in yellow. For each primary tumour the expression level of the specific gene is exhibited as red, if up-regulated and green, if down-regulated. Molecular subtypes of primary tumours are also demonstrated as; dark blue for luminal A, light blue for luminal B, yellow for HER2-like, pink for basal type and orange for normal-like tumours. *visceral metastasis: clinically evident visceral organ (liver, lung or brain) metastasis: yellow, absent; blue, present. **gene expression level: red, up-regulation; green, down-regulation. ***molecular subtype: dark blue, luminal A; light blue, luminal B; yellow, HER2-like; pink, basal type; orange, normal-like

Subsequently, we have performed supervised classification comparing 112 tumours from patients who developed visceral metastases and 39 tumours from patients who did not develop visceral metastases. Using this approach,14 differentially expressed genes were identified. The 14 genes included in the visceral organ specific gene expression signature were *WDR6*, *CDYL*, *ATP6V0A4*, *CHAD*, *IDUA*, *MYL5*, *PREP*, *RTN4IP1*, *BTG2*, *TPRG1*, *ABHD14A*, *KIF18A*, *S100PBP* and *BEND3* (Table 2). Figure 1 illustrates the heat map with gene expression pattern of these 14 genes in all tumours. Six of these genes, *CDYL*, *ATP6V0A4*, *PREP*, *RTN4IP1, BEND3* and *KIF18A* were up-regulated and the other genes were down-regulated. None of these genes overlapped with the genes included in the already published gene expression signatures for lung and brain metastasis [8, 10, 11]. Mapping to the Gene Ontology and Kyoto Encyclopaedia of Genes and Genomes databases revealed that five of these genes (ABDHD14A, IDUA, ATP6V0A4, PREP and KIF18A) were involved in hydrolase activity.

**Table 2:** The list of differentially expressed genes in visceral metastatic disease

	Accession number	HUGO	Description	*R*-value	*p*-value	Level of expression^a^
1	ILMN_1669484	WDR6	WD repeat domain 6	-0,319	6,50E-05	<
2	ILMN_1678075	CDYL	chromodomain protein, Y-like	0,316	7,84E-05	>
3	ILMN_1678186	ATP6V0A4	ATPase, H+ transporting, lysosomal V0 subunit a4	0,318	6,75E-05	>
4	ILMN_1700652	CHAD	chondroadherin	-0,319	6,42E-05	<
5	ILMN_1703041	IDUA	iduronidase, alpha-L-	-0,325	4,76E-05	<
6	ILMN_1746948	MYL5	myosin, light chain 5, regulatory	-0,313	8,97E-05	<
7	ILMN_1751887	PREP	prolyl endopeptidase	0,357	6,99E-06	>
8	ILMN_1758827	RTN4IP1	reticulon 4 interacting protein 1	0,314	8,84E-05	>
9	ILMN_1770085	BTG2	BTG family, member 2	-0,389	7,83E-07	<
10	ILMN_1790350	TPRG1	tumor protein p63 regulated 1	-0,336	2,41E-05	<
11	ILMN_1794213	ABHD14A	abhydrolase domain containing 14A	-0,321	5,86E-05	<
12	ILMN_2132161	KIF18A	kinesin family member 18A	0,341	1,81E-05	>
13	ILMN_2294274	S100PBP	S100P binding protein	-0,424	5,87E-08	<
14	ILMN_2375032	BEND3	BEN domain containing 3	0,316	7,91E-05	>

^a^ >, up-regulated; <, down-regulated

This 14-gene expression signature for visceral metastasis was subsequently validated in the training and the independent datasets. This novel signature was found to be positive in 72 primary tumours of the patients with metastatic breast carcinoma. Out of the 14-gene expression signature positive 72 patients 68 (94%) had visceral organ metastasis. Of 79 patients which were tested as negative for this signature, 35 (44.3%) did not develop visceral metastatic disease (*p* 2.13e− 08). Among the group of patients which had only visceral metastasis during the disease course (*n* = 18) 88.9% tested positive for the 14-gene expression signature (*p* 2.0e− 04). Among the ones which had visceral organ metastasis as first site of metastasis (*n* = 68) 70.6% tested positive for the signature (*p* 3.4e− 07).

When tested separately in ER-positive and ER-negative tumour groups, 50% of the ER-positive tumours and 60.5% the ER-negative tumours were assessed as visceral metastasis signature positive. Out of 54 ER+/signature + tumours 94.4% developed metastatic disease in a visceral organ; of 54 ER+ tumours/signature – tumours 53.7% did not develop visceral metastases (*p* 3.2e− 08). Of 24 ER−/signature+ tumours 91.7% had visceral organ metastasis and of 19 ER−/signature – tumours, 26.3% did not have a visceral organ metastasis (*p* 0.211).

Subsequently, the predictive value of 14-gene expression signature was investigated in an independent data set including 376 primary tumours of patients with metastatic breast carcinoma. Of 271 tumours assessed as visceral metastasis signature positive, 170 (62.7%) developed visceral organ metastases. Out of 105 tumours which were tested as negative, 66 (62,9%) had no evidence of metastatic disease to the visceral organs (*p* 9.68e− 06). This 14-gene expression signature was also assessed separately in ER-positive and ER- negative tumour groups (*n* = 373, ER status was missing in 3 cases). The 14-gene expression signature was found to be positive in 160 of the 245 ER-positive cases. 50% of these ER+/signature+ tumours developed visceral organ metastasis; of 85 ER+/signature – tumours 63.5% did not develop visceral metastases (*p* 4.50e− 02). There were 128 ER-negative tumours, 104 of which tested as positive for the visceral specific gene expression signature. 76% of these ER−/signature+ tumours developed visceral organ metastases; of 24 ER-negative tumours which were found to be negative for this signature 33.3% had no evidence of visceral organ metastasis (*p* 4.37e-01).

Table 3 summarizes the performance of visceral metastasis specific gene expression signature in the data sets described.

**Table 3:** Performance of the gene expression signature

Gene expression signature			Training data set			Independent data set		
		Signature	Visceral metastasis					
			yes	no	*p*	yes	no	*p*
14-gene expression signature^a^	All	present	68	4	*2.13e-08*	170	101	9.68e-06*
		absent	44	35		39	66	
	ER-positive	present	51	3	*3.2e-08*	80	80	4.50e-02**
		absent	25	29		31	54	
	ER-negative	present	22	2	*0.211*	79	25	4.37e-01***
		absent	14	5		16	8	

Abbreviations: ER, estrogen receptor

^a^The 14-gene expression signature developed in this study

*sensitivity: 81.3%, specificity: 39.5%

**sensitivity: 72.1%, specificity: 40.3%

***sensitivity: 83.2%, specificity: 24.2%

Univariate analyses in the training dataset revealed that next to development of visceral organ metastasis (ever, as first site of metastasis and as the only metastasis) the 14-gene expression signature was also found to be significantly correlated to the histologic subtype of the tumour, ER status, PR status and molecular subtype of the primary tumour (*p* 0.003, *p* < .001, *p* < .001, *p* < .001 and *p* < .001, respectively). The other parameters that were associated with development of visceral organ metastases were tumour size (*p* 0.002) tumour grade (*p* 0.009) and tumour type (*p* 0.008). Multivariate analyses showed that along with tumour type the 14-gene expression signature was remained significantly correlated to visceral organ metastasis (*p* 0.001, 95% CI 1.43–4.27).

Similarly, univariate analyses in the independent dataset showed that the 14-gene expression signature was significantly correlated with the development of metastatic disease to visceral organs (*p* < .001). Molecular subtype of the tumour, ER status, PR status and lymph node status were the other parameters that were statistically related to visceral metastasis in this data set. Multivariate analysis showed that hormone receptor status (ER and HER2) remained significantly correlated to the development of visceral metastases (*p* 0.047, 95% CI -3.36-0.003, ER status; *p* 0.025, 95% CI 0.2–3.2) whereas the 14-gene expression signature was not retained as a significant predictor (*p* 0.49, 95% CI -0.97-1.9).

Additional survival analyses in the training dataset exhibited that the 14-gene expression signature was associated with survival status of the patients, indicated by metastasis free survival and overall survival (*p* 0.001 and *p* < .001, respectively).

## Discussion

Development of visceral organ metastases in breast carcinoma is related to dismal prognosis with poor overall and metastasis free survival rates [17, 21]. The identification of genomic tumour characteristics associated with a higher likelihood of developing visceral organ metastasis will help understanding the mechanisms leading to the development of visceral metastases. In this study, we have compared the gene expression profiles of primary tumours of breast cancer patients who developed visceral organ metastasis to the ones without visceral metastasis using gene-expression microarrays. We have identified a unique group of genes which were differentially expressed in the group of tumours with clinical metastatic disease to the visceral organs. This association between 14- gene expression signature was significant not only with development of visceral organ metastasis (any time, as first site and as only site of metastasis) but also with both overall survival and metastasis free survival.

The identified gene expression signature included 14 genes, five (CDYL, *ATP6V0A4*, *PREP*, *RTN41P1, BEND3* and *Kif18A*) of which were up-regulated in the group of primary tumours of the patients with visceral organ metastasis. Two of these genes (*Kif18a* and *ATP6V0A4*) have been already reported to be associated with human breast carcinogenesis [22–25]. *Kif18a*, kinesin family number 18a, which has function to produce force and movement along microtubules, was previously found to be deregulated in different cancers including breast cancer [25]. It has been shown that overexpression of *Kif18a* is associated with tumour grade, development of metastasis and poor survival. Functional analyses have also shown that ablation of this protein results in inhibition of proliferative capability of breast cancer cells with inactivation of phosphatidylinositol 3-kinase-Akt signalling pathway [24]. *Zou* et al. have shown that knockdown of kinesin gene family members strongly disrupted the proliferation and induced the apoptosis in both tamoxifen-sensitive and resistant breast cancer cells and they have suggested the potential role of developing novel inhibitors of the kinesins for effective treatment of human cancers including tamoxifen-resistant breast cancer [25]. *ATP6VOA4*, vacuolar ATPase, H+ transporting, lysosomal V0 subunit a4, was another gene that found to be up-regulated in the group of tumours from patients with visceral metastatic disease. Previous studies have suggested an association between vacuolar ATPases (V-ATPase) and tumour invasion [26, 27]. Also, specific V-ATPase inhibitors, such as bafilomycin and concanamycin, have been shown to inhibit invasiveness of MDA-MB231 breast cancer cell lines [22, 23]. Subunit isoforms a3 and a4 are expressed at high levels in highly invasive breast cancer cell lines (MB231). It has been speculated that isoform a4 (*ATP6VOA4)* is involved in targeting V-ATPAse in cell membrane and this V-ATPase plays a role in invasive capability of these cells. This effect may be caused by locally acidifying the extracellular environment which may accelerate tumour invasion via creating an optimal acidic environment for proteases [28]. Further clinical studies investigating the effect of V-ATPase inhibitors on tumour invasiveness and metastasis will be of interest.

Nine out of 14 genes were downregulated in the subgroup of tumours with visceral organ metastasis. One of these downregulated genes is BTG2 (B- cell translocation gene-2), which has antiproliferative activity and has been reported to be altered in breast tumours [7, 29, 30]. It has also been shown that decrease of BTG2 expression in human breast cancer correlates with disease progression [31]. In order to explore the underlying mechanism, *Takahashi* and colleagues have further implemented experimental studies showing that knockdown of BTG2 expression led to increased cell motility. They have also demonstrated that BTG2 suppresses the activation of the HER2 pathway and suggested that HER pathway inhibitors, such as lapatinib, may play a role in controlling the progression of disease among breast cancers with decreased BTG2 expression. Moreover, the same group has demonstrated a modulator role of BTG2 on tamoxifen responsiveness in ER-positive/HER2-negative breast cancer and further validated BTG2 expression as a single predictor of survival following tamoxifen therapy [32]. Likewise, protein expression levels of BTG2 have also been found to be associated with 5-year overall survival in breast cancer patients. A prognostic model combining BTG2 expression, HER2 expression, patient age and Ki67 expression has been proposed with higher prediction accuracy than the currently used prognostic markers [33]. Consistent with the published data, we have shown that lower BTG2 expression was associated with shorter survival time (*p* 2.6e-04). Luminal type tumours had higher gene expression levels of BTG2 compared to HER2-like and basal type tumours (*p* 1.6e-10) and our current study has revealed a strong correlation between BTG2 expression and visceral metastasis (*p* 2.13e-08).

## Conclusions

With this study, we present a unique 14-gene expression signature for visceral metastasis in breast carcinomas. Further validation of this gene expression signature is warranted in order to test the reproducibility and the robustness of the correlations between the signature and metastatic behaviour.

## Additional file

Additional file 1: Illumina microarray data generated in this study. (XLSX 41 kb)

## Abbreviations

CT: Chemotherapy
ER: Estrogen receptor
HER2: Human epidermal growth factor receptor 2
HT: Hormonal therapy
MSS: Metastasis specific survival
OS: Overall survival
PR: Progesterone receptor
RECIST: Response evaluation criteria in solid tumours
